# Mammary Gland Development as a Sensitive End Point after Acute Prenatal Exposure to an Atrazine Metabolite Mixture in Female Long-Evans Rats

**DOI:** 10.1289/ehp.9612

**Published:** 2006-12-18

**Authors:** Rolondo R. Enoch, Jason P. Stanko, Sara N. Greiner, Geri L. Youngblood, Jennifer L. Rayner, Suzanne E. Fenton

**Affiliations:** 1 North Carolina Central University, Department of Biology, Durham, North Carolina, USA; 2 U.S. Environmental Protection Agency, Office of Research and Development, National Health and Environmental Effects Research Laboratory, Reproductive Toxicology Division, Research Triangle Park, North Carolina, USA; 3 University of North Carolina, School of Public Health, Department of Environmental Sciences and Engineering, Chapel Hill, North Carolina, USA

**Keywords:** atrazine, body weight, development, mammary gland, metabolites, mixture, prenatal exposure, puberty

## Abstract

**Background:**

Atrazine (ATR), a widely used chlorotriazine herbicide, inhibits a number of endocrine-dependent processes, including gonadotrophin surges and mammary gland development in rats. Chlorotriazine herbicides are rapidly metabolized in plants and animals to form a group of metabolites that are detected both in the environment and in exposed animals. The extent to which these metabolites are responsible directly for the observed health effects is not understood.

**Objectives:**

Our goal was to determine if a mixture of ATR metabolites, in proportions found in the environment, might produce developmental effects in Long-Evans rats following exposure late in pregnancy.

**Methods:**

We administered an ATR metabolite mixture (AMM) containing ATR, hydroxyatrazine, diaminochlorotriazine, deethylatrazine, and deisopropylatrazine orally to pregnant Long-Evans rats at 0.09, 0.87, or 8.73 mg/kg body weight (bw)/day, on gestation days 15–19, using 0 and 100 mg ATR/kg bw/day as negative and positive controls, respectively.

**Results:**

We observed no significant effect of acute AMM exposure on body weight gain in dams during the dosing period, weight loss in pups on postnatal day (PND)4, or pubertal timing, as is seen with ATR alone. However, as with ATR, we detected delayed mammary gland development, evaluated by whole mount analysis, as early as PND4 in all treatment groups.

**Conclusions:**

Our data suggest that acute exposure to AMM at levels as low as 0.09 mg/kg bw during late pregnancy causes persistent alterations in mammary gland development of female offspring, and that these effects do not appear to be related to bw or associated with pubertal timing.

Chloro-*s*-triazines, introduced in the late 1950s, include atrazine (ATR), simazine, propazine, terbutylazine, and cyanazine (Worthing and Walker 1987). These compounds have been heavily used as herbicides (Gysin and Knuesli 1960) and are commonly used to control growth of broadleaf weeds and grasses on crops such as corn, sugar cane, and sorghum [U.S. Environmental Protection Agency (EPA) 1997]. Chlorotriazines are frequently detectable in both ground and surface waters nationwide (Balu et al. 1998; U.S. EPA 2001; U.S. Geological Survey 1999; Wade et al. 1997), sometimes at levels exceeding the U.S. EPA maximum contaminant level of 3 ppb (U.S. EPA 2003b) for ATR.

ATR is catabolized by plants, microbes, and animals to yield four major metabolites (Figure 1): hydroxyatrazine (HA; 6-hydroxy-*N*-ethyl-*N*′-isopropyl-[1,3,5]triazine-2,4-diamine), diaminochlorotriazine (DACT; 6-chloro[1,3,5]triazine-2,4-diamine), deisopropylatrazine (DIA; 6-chloro-*N*-ethyl-[1,3,5]triazine-2,4-diamine), and deethyl-atrazine (DEA; 6-chloro-*N*-isopropyl-[1,3,5]triazine-2,4-diamine). Other chlorotriazines used in addition to or instead of ATR in the field, such as propazine and simazine, are similarly metabolized.

Because of their widespread environmental contamination in the United States, concern has been raised over the potential health effects these compounds may have on humans and wildlife species. Even after successful use restrictions, recent studies have detected ATR and its mercapturic acid derivatives in the urine of both farmers and nonfarmers, suggesting the general population is exposed to and excreting the herbicide (Curwin et al. 2005; Norrgran et al. 2006). Statewide groundwater monitoring and self-imposed minimum reporting levels for ATR and its chlorinated metabolites have been implemented in states growing a large percentage of their farm acreage in these crops [Illinois Department of Agriculture (IDA) 2006]. Reports indicate that about 18% of all samples of Illinois groundwater collected in 2002–2004 contained more than the minimum reporting levels (1.5 ppb) of one or more of the chlorinated ATR metabolites, yet few samples (< 2%) contained detectable ATR (IDA 2006).

ATR metabolites are relatively persistent in groundwater and soil/sediment, with mean aerobic and anaerobic soil half-lives ranging from 20–146 and 58–547 days, respectively, and aerobic aquatic half-life about 2-fold higher (Giddings et al. 2005). However, the half-life is much shorter in the rat. Approximately 85% of ATR-derived radioactivity, administered as ^14^C-labeled ATR, is excreted in the urine and feces of the rat in the first 72 hr after oral dosing (Bakke et al. 1972), with the majority of that excretion (85–95%) occurring in the first 24 hr.

Despite its relatively short half-life in the adult rat (< 1 day), many laboratories have shown that ATR administration adversely affects the endocrine system and reproductive tissue development in the rat (reviewed by Dooley et al. 2006). Long-term dietary exposure to 400 ppm ATR (~ 22.5 mg/kg) caused an early onset of mammary tumors in Sprague-Dawley rats, and the appearance of tumors was mediated by ATR-induced premature reproductive aging (Eldridge et al. 1994b, 1998, 1999a, 1999b). Yet, these findings have limited relevancy to human health because the condition of premature reproductive aging in the rat does not occur by the same process in women. Furthermore, the onset of rat mammary tumors was not increased after ATR-exposure in ovariectomized females (Eldridge et al. 1998).

Other pertinent developmental reproductive tissue effects have been demonstrated. ATR delays signs of puberty in both females and males when administered via oral gavage according to the “pubertal protocol,” described in the Endocrine Disruptor Screening Program (EDSP) Tier II screens (Laws et al. 2000; Stoker et al. 2000); it also causes full litter resorption in Fisher 344 rats (Narotsky et al. 2001). Other studies examined the effects of individual ATR metabolites on puberty in female and male Wistar rats (Laws et al. 2003; Stoker et al. 2002). These investigators found that DACT delayed puberty in female rats, and DIA, DEA, and DACT delayed puberty in males. They concluded that the molar equivalents of ATR’s chlorinated metabolites were as potent as ATR itself, suggesting that the metabolites themselves may mediate the effects of ATR seen in previous studies. Several studies have shown that ATR and its individual metabolites cause an alteration of normal patterns of ovarian and estrous cyclicity when administered to adult female Long-Evans, Fisher 344, or Sprague-Dawley rats (Cooper et al. 1996, 2000; Eldridge et al. 1994a, 1999b; Wetzel et al. 1994). Although ATR appeared to have antiestrogenic effects, it was also shown to have insignificant estrogen receptor binding and activation capability (Tennant et al. 1994).

A key ATR study (Cooper et al. 2000) showed that ATR (50–300 mg/kg by gavage) specifically inhibits the estrogen-induced surges of luteinizing hormone (LH) and prolactin (PRL) in ovariectomized Long-Evans and Sprague-Dawley rats, and therefore disrupts hypothalamic–pituitary–ovarian feedback loops. This study suggested the brain was the site of action for ATR, triggering further studies in the area of neuroendocrine action(s) of ATR (U.S. EPA 2003a). The current chronic reference dose (RfD; 0.018 mg/kg/day) is based on a 6-month rat feeding study using LH surge and estrous cycle alterations as the biomarkers of ATR’s potential to disrupt the hypothalamic–pituitary axis (U.S. EPA 2002). The acute RfD is > 5 times higher (0.1 mg/kg/day) and is based on the no observed adverse effect level (NOAEL) of 12.5 mg/kg/day (using a 100-fold safety factor) for diminished suckling–induced PRL surge (dam effect) and increased prostatitis in male Wistar rat offspring (Stoker et al. 1999). The only metabolite for which an acute RfD has been established is DACT (based on a developmental toxicity NOAEL of 2.5 mg/kg/day), the final breakdown product of the chlorotriazines (U.S. EPA 2002), yet there is no set maximum contaminant level for any of the chlorotriazine metabolites (U.S. EPA 2001).

After the acute ATR and DACT RfDs were established, Rayner et al. (2004) showed that a brief exposure to 100 mg ATR/kg bw *in utero* or via lactational ATR residuals delays female mammary gland development and vaginal opening (VO) in Long-Evans rats. In addition, the mammary effects in female offspring (delayed branching morphogenesis, fewer ducts) following acute prenatal exposure contribute to significantly decreased weight gain in an F_2_ generation without further exposure (Rayner et al. 2005). A 3-day exposure to ATR during late gestation was sufficient to cause these persistent effects. We do not currently know the mechanism by which ATR affects mammary gland development. Because ATR is rapidly metabolized in the body, it is possible that ATR metabolites mediate these effects. However, the effects of acute gestational exposure to ATR metabolites as a mixture (as they may occur in ground/surface water), or the individual metabolites, have not been examined at low doses.

The objective of these studies was to address the extent to which acute prenatal exposure to an ATR metabolite mixture (AMM; at relatively low doses) affects reproductive development of rat offspring. We specifically focus on the mammary gland as an outcome because it appears to be the reproductive tissue most sensitive to the effects of the mixture, at doses below the current acute developmental NOAEL for ATR of 6.25 mg/kg/day and the lowest observed adverse effect level of 12.5 mg/kg/day (U.S. EPA 2003a).

## Materials and Methods

### Animals

We obtained timed-pregnant Long-Evans rats (9–15 weeks of age; sperm positive = day 0) from Charles River Breeding Laboratories (Raleigh, NC). We housed females in clear plastic cages in a facility accredited by the Association for Assessment and Accreditation of Laboratory Animal Care. Cages contained heat-treated pine shavings (Beta Chips, North Eastern Products Inc., Warrensburg, NY), and animals were given food (Purina 5008 Rodent Chow; Ralston Purina Co., St. Louis, MO) and water *ad libitum*. They were maintained in a room with a 14:10-hr light cycle (lights out at 2100 hours) and treated humanely and with regard for alleviation of suffering, as required by the National Health and Environmental Effects Research Laboratory Institutional Animal Care and Use Committee.

### Dosing solutions

**Figure 1:**
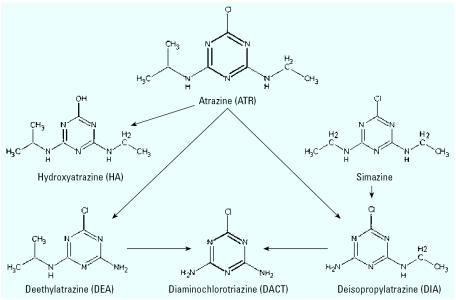
Structures of the environmental metabolites of two chlorotriazine herbicides, ATR, and simazine. ATR can be metabolized to HA, DIA, or DEA. The DEA and DIA forms are both quickly converted to DACT.

We prepared ATR and all metabolites (94.5–98.2% purity; Syngenta Crop Protection, Inc., Greensboro, NC) as a suspension in 1.0% methylcellulose (Sigma Chemical, St. Louis, MO) in distilled water. We formulated the AMM to contain ATR and its metabolites (Figure 1) in proportions and at levels reported in a survey of groundwater and surface water potentially contaminated by ATR (Balu et al. 1998; U.S. EPA 2001; U.S. Geological Survey 1999; Wade et al. 1997). Our AMM consisted of ATR (25%), HA (20%), DACT (35%), DEA (15%), and DIA (5%). We decided on the base AMM concentration (arbitrarily set at 250 ppm) using combined maximum ATR and metabolite concentrations detected in these sources. We based the mass given to the rats on an estimated 2 L/day water consumption of a woman wieghing 70 kg during her third trimester of pregnancy. We used adjusted molar equivalents to compare relative potency of the metabolites to ATR in future studies. Dose groups included 0 (1% methylcellulose vehicle), 0.09 (100 times the base AMM, 2.5 ppm), 0.87 (25 ppm), and 8.73 (250 ppm) mg AMM/kg bw/day, and 100 mg ATR/kg bw/day, which were delivered in a volume of 5.0 mL vehicle/kg bw. The 250-ppm dose calculations are shown in Table 1, and subsequent doses were made by serial dilution.

### Experimental design

**Figure 2:**
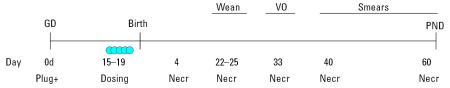
Overview of the study protocol. Timed-pregnant (plug+) Long-Evans rats (6–12 per treatment group per block, 2 blocks) were orally gavaged with twice-daily half-dose treatment of 0, 0.09, 0.87, or 8.73 mg AMM/kg bw/day or 100 mg ATR/kg bw/day on GD15–GD19. Female offspring were weighed at each necropsy (Necr) time point beginning at PND4 until the end of the study (PND60). Pups were weaned between PND22 and PND25, and onset of puberty was evaluated by day of VO beginning on PND28. Estrous cyclicity was monitored by smears beginning on PND42.

We conducted the study, as summarized in Figure 2, in two replicates (blocks) with five treatment groups per block (positive and negative controls present in each block). We oral gavaged pregnant Long-Evans rats (*n* ≥ 6/treatment/block) with 0, 0.09, 0.87 or 8.73 mg AMM/kg bw/day or 100 mg ATR/kg bw/day on gestation day (GD)15–GD19. We administered half doses twice daily (0700 and 1400 hours), adjusted for morning bw. We chose the 100-mg/kg dose to compare directly with results from our previous studies (Rayner et al. 2004, 2005). On prenatal day (PND)4, we weighed pups and equalized litters to six female pups and four male pups.

### Necropsy

We conducted a necropsy of female pups (2 pups/dam; *n* ≥ 5 dams, at each time) on PNDs 4, 25, 33, 40, and 60 (Figure 2). We used male pups in a separate study. Animals on PNDs 4, 33, 40, and 60 were decapitated, and PND25 animals were asphyxiated with CO_2_. We removed mammary glands (fourth and fifth) for whole-mount examination at all time points. We weighed, decapitated, and collected trunk blood from remaining females [*n* = 7–16 pups/treatment group (*n* ≥ 5 dams each)/block] on PND60. We removed and weighed the anterior pituitary, uterus, and ovaries devoid of fat. The contralateral mammary glands, ovaries, and uterus were fixed in 10% buffered formalin for histologic examination. We separated serum from the blood of female offspring and froze it at –80°C for measuring levels of thyroid-stimulating hormone (TSH), LH, and PRL by radioimmunoassays. We froze the anterior pituitary gland at –80°C for PRL and LH assays.

### Mammary whole mounts

In all experiments, we removed, fixed, and stained in carmine the fourth and/or fifth inguinal mammary glands as a whole mount, as previously described (Fenton et al. 2002). We examined whole mounts at 16× magnification on a Leica WILD M420 Macroscope (Leica Microsystems, Heerbrugg, Switzerland); two individuals without knowledge of treatment group scored them subjectively (scale of 1–4; 1 = poor development/structure, and 4 = normal development/structure). The specific criteria for scoring varied depending on the age of the group being evaluated, but they included the number of primary, secondary, and lateral branchings; the longitudinal growth toward the lymph node; budding patterns (or lack thereof); and the presence of terminal end buds or ducts. This scoring system has been previously shown to be associated with the actual numbers of branch points or terminal structures in rat mammary tissue (Fenton et al. 2002). We separated tissues (lacking identifying information) by developmental score as they were evaluated, compared each tissue within a score for consistency, recorded data, and then translated unique identifying information. We captured images of glands representative of the mean score of the group using a Photometrics CoolSNAP camera running RSImage 1.9.2 (Roper Scientific, Inc., Tucson, AZ) for Windows (Microsoft Corporation, Redmond, WA).

### Histology

We removed the fourth inguinal mammary gland on PND60 for histologic assessment of terminal structures. We compressed the glands in cassettes in two changes of 10% buffered formalin for 48 hr each, at room temperature. We then transferred fixed glands to 70% ethanol, processed them in paraffin, cut them into 5-μm sections, and stained them in hematoxylin and eosin. On PND60, we also fixed the ovaries and uterus in formalin for at least 48 hr. These tissues were rinsed and stored in 70% alcohol until embedded in paraffin, sectioned (5 μm), and stained with hematoxylin and eosin. We viewed all sectioned tissues by light microscopy on a Leitz Laborlux microscope (100× magnification; E. Leitz, Leica Microsystems) for any detectable abnormalities.

### Vaginal opening and estrous cyclicity

Beginning on PND28, we observed all female offspring in this study surviving past weaning for VO, assessed by the presence of a fully opened vagina, and recorded weight at VO. Vaginal smears (as described by Cooper et al. 1999) were conducted beginning on PND42, for the animals surviving past PND40, and continued until PND60. We classified the vaginal smears as described by Everett (1989) and defined extended estrus as exhibiting cornified cells with no leukocytes for ≥ 3 days and extended diestrus as the presence of leukocytes for ≥ 4 days (Cooper et al. 1999).

### Radioimmunoassays

We analyzed serum samples from 60-day-old offspring euthanized between 0800 (1 hr after lights on) and 1500 hours (6 hr before lights out), in the diestrus stage of the estrous cycle, for PRL, LH and TSH, and we analyzed anterior pituitaries for PRL and LH by radioimmunoassay, as previously described by Cooper et al. (2000). We moved all animals to holding rooms the day before necropsy and euthanized them as rapidly as possible to minimize the stress effect of movement and handling on hormone levels. We performed the assays using the following materials supplied by the National Institute of Arthritis, Diabetes, Digestive, and Kidney Diseases for LH, PRL, and TSH, respectively: iodination preparation I-10, I-6, I-9; reference preparation RP-3, RP-3, RP-3; and antisera S-11, S-9, S-6. The iodination preparation was radiolabeled with Na^125^I (Dupont/New England Nuclear, Boston, MA) by a modification of the chloramine-T method of Greenwood et al. (1963). We serially diluted standard reference preparations for the standard curves. Serum samples and anterior pituitary homogenates were diluted (as appropriate) to a final volume of 500 μL in 100 mM phosphate buffer, containing 1% bovine serum albumin (BSA). We ran quality control standards at the beginning and end of each assay. Assay tubes containing 200 μL primary antisera in 100 mM potassium phosphate, 76.8 mM EDTA, 1% BSA, and 3% normal rabbit serum were vortexed and incubated then at 4°C for 24 hr. We added 100 μL iodinated hormone to each tube, vortexed the contents, and incubated the tubes for 24 hr. We then added secondary antibody (goat anti-rabbit gamma globulin, 1 unit/100 μL; Calbiochem, San Diego, CA), vortexed the tubes, and incubated them for 24 hr. We centrifuged the samples at 1,260 × *g* for 30 min, aspirated the supernatant, and counted the sample tube, with pellet, on a gamma counter. We performed the assays in duplicate, and all assay coefficients of variation were < 10%. We adjusted data for sample dilution and volume.

### Fetal weight study

We conducted these studies exactly as described in Rayner et al. (2005). Timed-pregnant Long-Evans dams were treated with 0 or 8.73 mg AMM/kg bw (*n* = 8 dams/treatment) on GD15–GD19 and euthanized on GD20. We removed the litters from the dam and recorded the sex and weight of each fetus. One control dam died during the study.

### Statistical analysis

We used dam as the unit of exposure (reported findings as dam means) for all analyses. We analyzed data for effects by analysis of variance using the least-squares means or mixed models in SAS (SAS Institute, Inc. Cary, NC); *p* < 0.05 indicates statistical significance. We assessed replicate (block) effects for each parameter. Organ weights were analyzed using both the body weight at necropsy and the dam as a covariate (mixed model). We compared experimental groups with the control group and with each other (Dunnett’s) and performed categorical data analyses on cyclicity data. Data are reported as mean ± SE except where noted.

## Results

### Maternal weight gain

**Figure 3:**
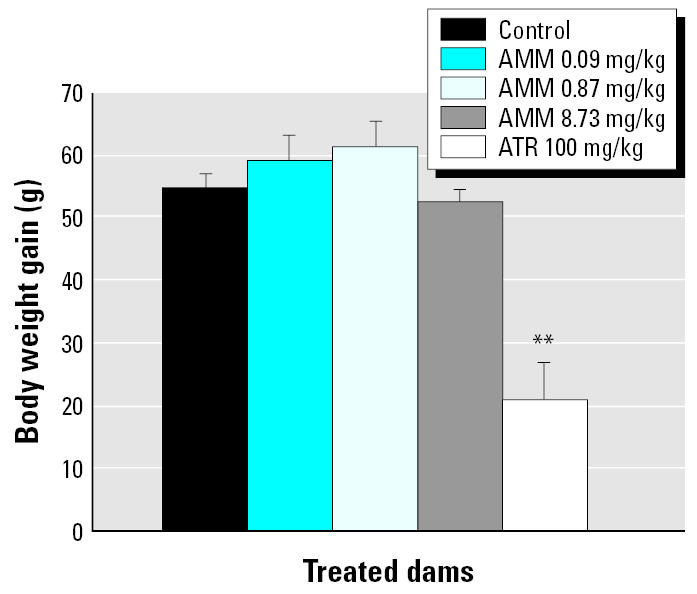
Body weight gain (g) in control rat dams or those treated with ATR or AMM during the dosing period (GD15–GD19). Data are presented as mean ± SE (*n* = 15–20 dams/group). **p < 0.01 compared with controls.

Maternal weight gain was significantly reduced during the dosing period (less than half of control value) after ATR-only exposure (*p* < 0.05). However, we found no reduction in weight gain during that period in the AMM-treated dams (Figure 3). In fact, dams in the lower two AMM dose groups exhibited a slight increase in weight gain (7–12% more than controls).

### Offspring body weights

Rayner et al. (2004) reported that *in utero* exposure to 100 mg ATR/kg bw during GD15–GD19 reduced pup weight by 6–7% on PND4. However, in the present study, female offspring prenatally exposed to 100 mg ATR/kg bw were not statistically different in body weight from control pups (Table 2) at any age evaluated (data not shown). At PND4, the mean offspring weight of ATR exposed pups was 3% less than control pups, which is consistent with the 6% decrease seen in our previous study (Rayner et al. 2004). Although the AMM contained 25% ATR, it significantly increased the mean bw at PND4 over that in control pups by 7–8% at all doses tested (*n* = 6–12 pups/treatment/block, *p* < 0.01; Table 2).

We weighed female offspring at several time points after PND4. We found no effect of treatment on mean bw in female offspring at PND25, PND33, or PND40 (data not shown). However, on PND60 female offspring of dams treated with 0.87 and 8.73 mg AMM/kg bw weighed significantly more than controls (*p* < 0.05; Table 2). Some of these effects on bw may be attributed to random selection of offspring at the individual time points, but all offspring were weighed at PND4. To determine whether the difference in weight of PND4 AMM pups originated from prenatal exposure or was due to smaller litter sizes in treated dams (before equalizing litter size), we conducted a separate study to evaluate the effects of AMM exposure on fetus number and fetal body weight at GD20, the day after the dosing ended. In this study, dams exposed to 8.73 mg AMM/kg bw had numbers of pups similar to controls (12.1 ± 1.4 in the AMM litter and 13.7 ± 1.0 in the control litter; *n* = 7–8 dams/treatment group), and no dead fetuses were observed in any treatment group. The weights of both male and female fetuses of AMM-treated dams were significantly higher than those of control dams on GD20 (Table 3). These data demonstrate that the apparent increased weight gain of AMM-exposed offspring present on PND4 stems from increased fetal weight. These data suggest that the doses of AMM and ATR used in this study have opposing effects on maternal and postnatal offspring body weight gain.

### Vaginal opening

**Figure 4:**
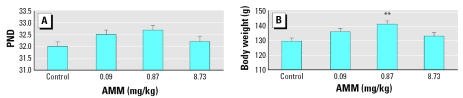
Effect of AMM on day of VO (*A*) and weight at VO (*B*). Data are presented as mean ± SE; *n* ≥ 10 pups/treatment. **p < 0.01 compared with controls.

We assessed the onset of puberty of the female offspring by VO. The day of VO for AMM-treated animals, regardless of dose, was similar to that of controls (Figure 4A). However, when comparing the weights of pups on their respective day of VO, we found an effect of AMM exposure on bw. The animals in the 0.87-mg AMM/kg bw group were significantly heavier than control offspring (*p* < 0.001) at the time of VO, as were the females in the 0.09-mg/kg AMM group (*p* = 0.052; Figure 4B).

### Mammary gland development

**Figure 5:**
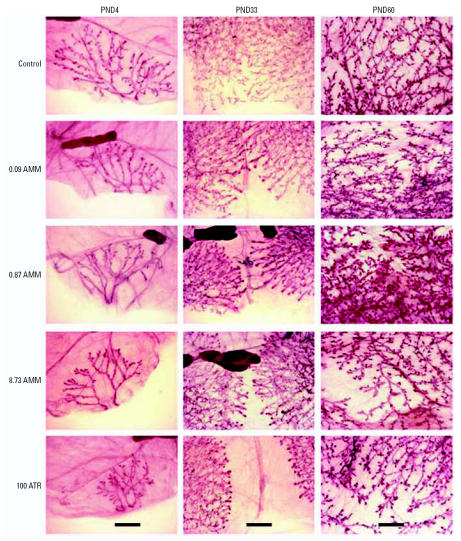
Mammary gland whole mounts from female offspring prenatally exposed to AMM or ATR (mg/kg bw/day). Images (at 16 × magnification) represent the mean scores for each treatment and age as indicated in Table 4, and illustrate significant changes with increased AMM exposure, such as fewer and smaller ductal buds and lateral branching (PND4); undifferentiated end buds and glands not grown together (PND33); and TEBs, large lobuloaveolar units, and immature branching (PND60). Large dark structures present in a few segments are stained lymph nodes. Bars = 2 mm.

We compared the carmine-stained mammary epithelia of AMM- and ATR-exposed animals to that of controls by whole-mount analysis on PND4, at weaning, and on PND33, PND40, and PND60, denoting the developmental score within an age group after comparison of all slides receiving a similar score (as described in “Materials and Methods”). We found a significant effect of AMM exposure on growth and development of glands; significant delays were seen at each of these time points except PND33 (Table 4; development in the PND33 control group was unusually poor compared with that of historical controls); these developmental delays were consistently detectable in whole mounts of the fourth and fifth inguinal glands (Figure 5). At weaning, PND40, and PND60 the glands of all exposed females were significantly different from those of controls (*p* < 0.0001). This was also true at PND4 for the 0.87-mg AMM/kg bw and ATR exposure groups (*p* < 0.01) and at PND33 for the 8.73-mg AMM/kg bw and ATR exposure groups (*p* < 0.05). The AMM and ATR-exposed animals at PND4 had delayed migration of the epithelium through the mammary fat pad and limited lateral branching from the primary and secondary ducts compared with controls. Typically, at PND33, glands 4 and 5 have grown together, and differentiation of terminal end buds (TEBs) is apparent in control animals. However, in this group of control animals, that was not the case, causing the average control score to be lower than that previously observed in this strain of rat (Fenton et al. 2002; Rayner et al. 2004, 2005). The control glands at PND4 were also not as well developed as expected.

The effects of the AMM exposure were persistent. Although TEB had differentiated and lobules were regressing in control animals at PND60 (maturity), differentiation within the fat pad of exposed animals was hindered as treatment dose was increased. This was particularly evident in the glands of 8.73-mg AMM/kg bw and ATR-exposed animals (Figure 5). The mammary gland developmental scores were equivalent in those two dose groups over time (even though there is a > 10-fold difference in total dose). No histopathologic lesions (dysplasia) were noted in the mammary glands of these animals; we confirmed the presence of TEB at PND60 in AMM-exposed animals via histology (not shown).

### Reproductive tract and cyclicity evaluation

Organ weights of sexually mature female offspring evaluated in diestrus are shown in Table 5. We analyzed these data by treatment group and weighted the data by dam and bw using a mixed-model approach. There was a significant effect of AMM exposure on pituitary and ovary weights (*p* < 0.05). The pre-natally AMM-exposed females demonstrated a 14–19% increase in pituitary weight at PND60. However, ovary weight was significantly increased only in offspring exposed to 0.09 mg AMM/kg bw (*p* < 0.01), whereas the uterine weight of ATR-exposed females was decreased slightly compared with controls.

We examined the uteri from diestrus animals microscopically for lesions/abnormalities or inflammation. We detected no differences for AMM or ATR-treated females compared with either controls or each other (data not shown). We also microscopically evaluated both ovaries from each sexually mature female to ensure that normal follicular and luteal structures were present. We detected no differences between AMM-treated, ATR-treated, or control ovaries (data not shown). In agreement with these histopathologic results, there was no significant effect of treatment on early estrous cyclicity (data not shown). The majority of animals in all groups were cycling normally. In all treatment groups (*n* = 7 controls; *n* = 10–20/treatment group) at least one animal displayed a single prolonged diestrus or estrus event, or had an abnormal cycle length. However, in the ATR-exposed group, 4 of 10 females had cycles ≥ 6 days long. This was also seen in our previous studies (Rayner et al. 2004).

### Hormone analysis

Hormone concentrations from 60-day-old offspring in the diestrus stage of the estrous cycle are shown in Table 6. We found a significant effect of treatment on pituitary PRL concentrations. The animals exposed to 0.87 mg AMM/kg bw and ATR were significantly different from control animals (*p* < 0.05 and *p* < 0.01, respectively). Although the ATR-induced serum PRL levels were only about 40% of those found in controls, we detected no other significant effects of treatment on serum or pituitary hormones in the study (including TSH, not shown).

## Discussion

This study is the first to report exposure of rodents to a mixture of ATR and its environmental metabolites. We report effects on reproductive tissue development following acute gestational exposure to relatively low doses of ATR metabolites in the rat. The data demonstrate that an acute 5-day exposure to AMM during late pregnancy has a significant impact on mammary growth and development, with delayed development prominent as early as PND4 and effects evident in all treatment groups at weaning. Fetal mammary development has previously been shown to be sensitive to high-dose ATR (Rayner et al. 2004, 2005), as well as other environmental contaminants (Fenton et al. 2002; Johnson et al. 2003; Markey et al. 2001; White et al. 2006). However, this same low dose of AMM did not impact onset of VO (a typical indicator of puberty in rodents) or significantly reduce mean bw in the offspring or treated dams. Importantly, the lowest AMM dose used in our study (0.09 mg/kg bw), containing only 25% ATR (by weight), caused persistent delays in mammary development that were still evident at PND60. These levels of ATR metabolites are below the current acute developmental NOAEL for ATR of 6.25 mg/kg/day. In previous work (Rayner et al. 2005), delays in mammary development into adulthood (such as those we found on PND60) resulted in adverse effects on weight gain of F_2_ pups without further exposure, possibly due to insufficient milk production of the F_1_ ATR-exposed dams’ underdeveloped mammary glands. The long-term impact of mammary effects from this low-dose mixture is not yet known.

Previous studies in female Wistar rats have shown that extended peripubertal exposure to ATR at doses as low as 50 mg/kg bw/day (PND21–PND41) delayed puberty (Laws et al. 2000). Delayed VO in Long-Evans rats has been consistently observed with late gestational and/or residual lactational ATR exposure (Rayner et al. 2004, 2005). However, unlike ATR, our data demonstrate no significant effect on VO from the low-dose AMM–exposed females. Our data, showing perturbation of mammary development in female offspring after prenatal AMM exposure well before the hormones of puberty have begun to increase (PND4 and PND22), emphasize that mammary gland development and VO may not be regulated by similar signaling pathways or modes of action. Further, mammary gland development is more sensitive to the effects of ATR and its metabolites than is VO. This is consistent with effects observed following lactational and *in utero* exposure to ATR, where we have shown that GD15–GD19 is a critical period for growth and development of the mammary glands but not for other female reproductive tissues (Rayner et al. 2004, 2005). This sensitivity could arise because mammary gland growth and development may be directly responsive to these compounds (e.g., more compound burden in this tissue), the assay for discerning the effects may be considerably more sensitive (whole mount analysis vs. VO), or the latter part of gestation and early lactation (time of exposure) are sensitive periods for mammary gland development, but not for VO timing.

Although there are no other published studies that have evaluated short gestational exposures to ATR and its metabolites, Stoker et al. (2002) have shown that exposure to individual ATR metabolites in male Wistar rats treated for the entire peripubertal period (PND23–PND53) caused a decrease in mean bw of the animals, as well as a delay in puberty. In the present study, we found no indication of reduced bw in females exposed to AMM (pups or dams), but a significant increase in bw was noted on PND4 and PND60. In Wistar rats, no ATR-induced effects on puberty were seen at doses < 50 mg/kg bw/day (Laws et al. 2000). Because our highest dose of AMM (8.73 mg/kg bw/day) contains only 25% ATR (1.79 mg/kg bw/day), we did not expect to observe a change in the timing of puberty. However, all doses of AMM affected the mammary and anterior pituitary glands. The anterior pituitary gland may also be sensitive to the effects of exposure to AMM because the significant increases in weight of this tissue following exposure to AMM are reminiscent of the significant changes seen on PND58 in a previous study (Rayner et al. 2004) using 100 mg ATR/kg bw on GD15–GD19.

Our data [the present study and Rayner et al. (2004, 2005)] indicate that mammary gland growth and development in the female appear to be significantly affected by low-level AMM and ATR exposure *in utero*. It has been previously suggested that decreased body weight is correlated with decreased or delayed mammary gland development in virgin mice (Engelman et al. 1994) and that toxicant-induced decreases in bw can cause delayed puberty (Stoker et al. 2000). However, we observed no decrease in mean bw or hormone levels, or delay in onset of puberty that coincides with the developmental delays of the mammary gland. Interestingly, our data demonstrate a gain in bw in AMM-exposed fetuses as early as GD20. Delayed mammary growth and development following deletion of genes known to be involved in puberty and growth has also been shown in various knockout mouse lines, implicating changes in puberty or endocrine hormone levels as possible mechanisms (Walker and Korach 2004). We detected little change in serum or pituitary hormone levels between controls and offspring exposed to ATR or AMM *in utero*. In the female offspring, pituitary PRL concentrations were significantly higher in both ATR and mid-dose AMM-exposed groups than in controls. However, all of our measured values appear to be elevated beyond the normal physiologic concentrations of about 1,006 ± 104 ng/mg tissue (Cooper et al. 1999), and may be typical for young female rats (60 days of age vs. 90 or 120 days of age).

In conclusion, acute administration of the mixture of ATR and its metabolites during gestation had a significant effect on mammary growth and development in female offspring and these effects persisted into adulthood. The doses of AMM necessary for these effects were > 10-fold lower than doses of ATR previously shown to induce mammary developmental delays. This finding brings attention to the fact that the metabolites of the chlorotriazines deserve attention. The fate and elimination kinetics of the mixed ATR metabolites may differ from those of ATR alone, resulting in these low-dose effects. It is also possible that a single metabolite is responsible for these effects, or that ATR or one of the metabolites present in the AMM persists for a longer time in the pregnant and lactating rat, theoretically extending the actual exposure period.

Studies are under way to investigate the particular metabolite—or combination of metabolites—responsible for the effects observed in the present study; also, studies are being conducted to address whether these are direct effects on mammary cells or an indirect effect. Further studies into the critical elements of neonatal mammary gland development are necessary to clarify the effects of these low-dose (particularly at 0.09 mg/kg), short-term exposures to ATR and its metabolites. Once a mechanism of action for chlorotriazine metabolites on mammary tissue has been determined, it will be important to consider the many similarities between mechanisms regulating growth and differentiation in human and rat mammary glands [such as timing of appearance of mammary structures and bud outgrowth (Fenton 2006)] in order to determine the potential relevancy of these findings with respect to human health.

## Figures and Tables

**Table 1:** Formulation of AMM.

Compound	FW	AME	Compound in AMM (%)	Compound in AMM (ppm)^a^	AME (adj ppm)^b^	Adj ppm (%)^c^	Conc in stock (mg/kg)^d^
ATR	215.7	1	25	62.5	62.5	20.5	1.786
DACT	145.5	1.482	35	87.5	129.7	42.5	3.706
HA	197.2	1.094	20	50.0	54.7	17.9	1.563
DEA	187.7	1.149	15	37.5	43.1	14.1	1.231
DIA	173.7	1.242	5	12.5	15.5	5.1	0.443
Total			100	250.0	305.5	100	8.729

Abbreviations: Adj, adjusted; AME, adjusted molar equivalent of ATR (ATR FW/metabolite FW); Conc, concentration; FW, formula weight. 8.73 mg AMM/kg bw = 250 ppm.

a The ppm value of % in AMM.

b AMM % as ppm adjusted to reflect the AME.

c Compound % of AME adjusted ppm.

d Amount in the highest mixture dose. Assuming 2-L consumption and 70-kg bw for a human, [AME adj ppm (as mg)/L] ×(2L/day) × (1/70 kg) = mg/kg/day.

**Table 2:** Effect of AMM and ATR (mg/kg) on bw (g) of female offspring.

	Control	0.09 AMM	0.87 AMM	8.73 AMM	ATR
PND4	10.03 ± 0.13	10.80 ± 0.16#	10.77 ± 0.17**	10.69 ± 0.17**	9.68 ± 0.21
PND60	252.7 ± 2.0	254.5 ± 4.8	271.8 ± 6.4*	269.0 ± 5.2*	262.8 ± 7.1

Data are presented as mean ± SE; *n* ≥ 10 pups/treatment.

* *p* < 0.05,

** *p* < 0.01, and

# *p* < 0.001, compared with controls.

**Table 3:** Effect of gestational exposure to AMM on litter size and fetal bw.

	Control	AMM (8.73 mg/kg)
Live fetuses/litter	13.71 ± 1.04	12.13 ± 1.36^a^
Resorptions/litter	0	0.75 ± 0.25
F fetus bw (g)	1.97 ± 0.03	2.14 ± 0.03**
M fetus bw (g)	2.00 ± 0.03	2.19 ± 0.03**
F + M fetus bw (g)	1.99 ± 0.02	2.16 ± 0.02**

Abbreviations: F, female; M, male. Data are presented as mean ± SE; *n* = 7 litters for control, *n* = 8 litters for AMM.

a No dead fetuses.

** *p* < 0.01 compared with controls.

**Table 4:** Effects of AMM (mg/kg bw/day) on mammary gland scores.

PND	Control	0.09 AMM	0.87 AMM	8.73 AMM	ATR
4	3.27 ± 0.28	2.52 ± 0.18	2.00 ± 0.39**	2.47 ± 0.18	2.03 ± 0.31**
25	3.83 ± 0.08	2.87 ± 0.11#	2.50 ± 0.07#	2.55 ± 0.12#	2.10 ± 0.28#
33^a^	2.50 ± 0.25	2.28 ± 0.23	2.15 ± 0.22	1.75 ± 0.21*	1.70 ± 0.13
40	3.40 ± 0.11	2.32 ± 0.16#	2.13 ± 0.11#	1.83 ± 0.10#	1.93 ± 0.17#
60	3.25 ± 0.05	2.39 ± 0.13#	2.06 ± 0.11#	1.81 ± 0.05#	1.75 ± 0.08#

Data are presented as mean ± SE; *n* ≥ 10 pups/treatment. Score: 1 = stunted growth pattern; 4 = normal growth for age.

a Unusually poor development in the control group compared with historical controls.

* *p* < 0.05,

** *p* < 0.01, and

# *p* < 0.001 compared with controls.

**Table 5:** Effect of AMM (mg/kg bw/day) on female organ weight.

	Control	0.09 AMM	0.87 AMM	8.73 AMM	ATR
Pituitary (mg)	9.1 ± 0.4	10.8 ± 0.3**	10.4 ± 0.3**	10.6 ± 0.4**	9.6 ± 0.5
Ovary (mg)	106.6 ± 3.6	131.0 ± 5.9**	112.7 ± 4.4	121.1 ± 3.9	114.2 ± 5.5
Uterus (g)	328.6 ± 13.8	317.7 ± 12.0	352.8 ± 24.1	311.8 ± 19.7	292.6 ± 16.0

Data are presented as mean ± SE; *n* ≥ 10 pups/treatment. Significant effects of treatment were observed using pup bw as a covariate.

** *p* ≤0.01 compared with controls.

**Table 6:** Effect of AMM (mg/kg bw/day) on female hormone levels at PND60.

	Control	0.09 AMM	0.87 AMM	8.73 AMM	ATR
sPRL (ng/mL)	10.10 ± 1.86	7.21 ± 1.28	9.21 ± 2.16	8.79 ± 3.98	3.83 ± 0.72
pPRL (ng/mg)	1,443 ± 101	1,595 ± 86	2,045 ± 116**	1,431 ± 94	2,052 ± 427*
sLH (ng/mL)	0.289 ± 0.030	0.343 ± 0.052	0.397 ± 0.138	0.377 ± 0.086	0.331 ± 0.066
pLH (ng/mg)	885 ± 45	924 ± 57	1,039 ± 81	763 ± 50	755 ± 46

Abbreviations: p, pituitary gland; s, serum. Data are presented as mean ± SE; *n* ≥ 10 pups/treatment.

* *p* < 0.05, and

** *p* < 0.01 compared with controls.
